# Carers’ interpretation of the recall period and perspective-taking when completing the EQ health and wellbeing instrument (EQ-HWB)-9 as proxies for people with dementia: a think-aloud study

**DOI:** 10.1007/s11136-026-04230-y

**Published:** 2026-04-01

**Authors:** Carrie-Anne Ng, Kathleen Doherty, Margo Bryan, Jill Carlton, Tim Luckett, Brendan Mulhern, Richard Norman, Karen Wills, Jessica Roydhouse

**Affiliations:** 1 Centre for Health Economics Research and Evaluation, University of Technology Sydney, Sydney, NSW Australia; 2 Wicking Dementia Research and Education Centre, University of Tasmania, Hobart, TAS Australia; 3 Lived Experience Partner, Hobart, TAS Australia; 4 Sheffield Centre for Health and Related Research, University of Sheffield, Sheffield, UK; 5 Improving Palliative, Aged and Chronic Care Through Clinical Research and Translation (IMPACCT), Faculty of Health, University of Technology Sydney, Sydney, NSW Australia; 6 School of Population Health, Curtin University, Perth, WA Australia; 7 Menzies Institute for Medical Research, University of Tasmania, 15-17 Liverpool Street, Hobart, TAS 7000 Australia

**Keywords:** Proxy, Dementia, Well-being, Recall, Perspective

## Abstract

**Purpose:**

Patient-reported outcome measures are often administered with specific recall periods to standardise reporting. However, research on recall periods in proxy reporting is limited. This study sought to explore (1) how informal carers interpret the “last 7 days” recall period of the EQ health and well-being instrument (EQ-HWB)-9 and (2) the perspective they adopt when completing the measure to report on the health and well-being of persons with dementia.

**Methods:**

A qualitative, descriptive study was conducted. Convenience sampling was used for recruitment. We interviewed informal carers of persons with dementia in Australia using semi-structured cognitive interviews. Participants were asked to think aloud when completing the EQ-HWB-9, and three additional positively-framed questions from the EQ-HWB. Thematic analysis was used, where transcripts were inductively coded to capture new insights and deductively coded as guided by the EQ-HWB-9 questions.

**Results:**

Nineteen carers completed interviews. Carers used two main retrieval strategies: supplementing recent observations with key events or self-report occurring slightly outside the recall period, and drawing on the past to contextualise recent behaviours. All participants used their own observations and opinions of the person with dementia (“proxy–proxy perspective”). When questions about the person’s feelings were difficult to answer, carers relied on various observable behaviours as indirect indicators. Nearly half also imagined how the person might answer (“proxy–patient perspective”), even if they sometimes disagreed with the person’s self-assessment.

**Conclusion:**

The 7-day recall period for the EQ-HWB-9 was more consistently adhered to for observable aspects of health. Wording changes may be needed to support consistent use, particularly for less observable items. Challenges in perspective-taking and adherence were also identified, suggesting areas to address in instructions.

**Supplementary Information:**

The online version contains supplementary material available at 10.1007/s11136-026-04230-y.

## Abstract (plain language summary)

Questionnaires are often used to measure patients’ health and experiences. These questionnaires usually ask people to think about a specific time frame—called a “recall period”. Sometimes, people living with dementia cannot complete these questionnaires themselves, so someone else (“proxies”), like their carer, may be asked to answer on their behalf. We are not sure how proxies think about and use the recall period when answering. This matters because using a time frame helps us collect consistent information, which is important for comparisons between people and tracking health changes over time. If people use different time frames when responding to the same question, it may be hard to interpret the results.

In this study, we interviewed carers of people living with dementia about how they used the “last 7 days” recall period in a questionnaire (EQ-HWB-9) about the person’s health and wellbeing. We found that carers sometimes included observations that happened outside the last 7 days if they felt these were important. Carers also compared recent behaviours to how the person acted in the past. All carers based their answers on their own observations of the person living with dementia (“proxy–proxy perspective”), and when it was hard to judge feelings, they looked to behaviours as clues.

These findings show that providing clear instructions may be needed for carers to stay within the recall period, and highlights helpful areas to research in the future.

**Supplementary Information:**

The online version contains supplementary material available at 10.1007/s11136-026-04230-y.

## Introduction

A patient-reported outcome (PRO) is defined as a report provided directly by a patient about their health or function, without interpretation by anyone else, such as a clinician [1]. Regulatory agencies prioritise patient self-report of PROs and generally discourage the use of reports from others, such as proxies [1, 2]. Nonetheless, proxy reporting plays an important role in many health contexts, including paediatrics [3], palliative care [4], and dementia [5]. Despite the widespread use of proxy reporting, there are substantial gaps relating to the development and subsequent use of measures for proxy report [6].

In some cases, proxy-reported measures are generated through simple adaptations of PRO measures (PROMs), for example by changing item wording from ‘I feel’ to ‘the patient feels’. Such measures are used in a way for which they were not originally designed, and without supporting psychometric evidence, may not be appropriate for proxy-report. However, the focus of most research on proxy reporting has been on concordance, or the consistency between patient and proxy reports for the same individual, using the same measure. Evaluation of concordance alone is unlikely to provide sufficient psychometric evidence for a given measure [6]. Furthermore, even if a measure is adapted simply, proxies and patients may interpret the measure differently [7]. Measurement invariance is a form of validity evidence [8], and there are some studies exploring the issue of differential item functioning (DIF) between proxies and patients [7, 9, 10], though infrequent compared to concordance studies.

Another example of a gap in measurement research relating to proxies is the recall period. The assessment of a recall period is part of the assessment of content validity [11], which is one of the most important measurement properties [12]. Recall periods for PROMs have been the subject of considerable research [13–16], and various considerations for selecting these have been put forth [17, 18]. In contrast, the evidence base for recall periods in proxy-reported measures is less well-developed. Recent reviews on proxy-reported measures for both adult [6] and paediatric [19] populations have not addressed this issue. A review on optimal recall periods noted this, but anticipated that considerations relevant to patient-report may also apply to proxy reports [18]. However, a critical difference is that proxy reporters, by definition, have less information about patients compared to patients themselves [20]. As a result, proxies and patients/self-respondents appear to use different approaches when answering questions [21, 22]. Thus, it is unclear to what extent findings about recall periods for PROMs can be extrapolated to proxy-reported measures.

Across the broader proxy literature, evidence regarding the optimal recall period has been mixed. For example, the use of a 2-week, rather than a 1-week, period resulted in an underestimation of diarrhoeal prevalence in children [23]. Similarly, 2-week recall periods underestimated disease symptoms and healthcare utilisation in children, compared to shorter periods of 3 days [24]. When considering the trade-off between sample size requirements and measurement bias in prevalence estimates, 7-day recall periods have been suggested for proxy-reported illness in children [25]. It is unclear how widely these recommendations can be extrapolated as these studies involved adult caregivers reporting on behalf of children rather than adults. Additionally, this evidence largely pertains to reporting on the occurrence of specific events (i.e., symptoms, illnesses, healthcare utilisation), and it is not clear how generalisable this finding may be to other domains such as health-related quality of life (HRQoL) and well-being.

Furthermore, a measurement issue specific to proxies relates to the different viewpoints or perspectives a proxy may adopt when reporting on the patient’s behalf. Such perspectives have been incorporated in regulatory definitions of proxy reporting; for example, the European Medicines Agency (EMA) defines a proxy as “a person who reports an outcome as if she/he was the patient him/herself” [2], and the US Food and Drug Administration (FDA) similarly states that a proxy reports “as if he or she were the patient” [1]. However, the literature, including Pickard and Knight’s seminal work (2005), recognises that proxies may adopt more than one perspective [26]. Specifically, Pickard and Knight [26] distinguish between the *proxy–patient* perspective (i.e., reporting as if the proxy is the patient) and the *proxy–proxy* perspective (i.e., reporting from the proxy’s own perspective). Some measures with proxy versions offer the option to choose between these perspectives, such as ASCOT [27] and the EQ-5D [28], whereas others such as DEMQOL and QOL-AD only offer the proxy–patient perspective [29].

The question of which perspective may be better in minimising the difference in reporting between patients and proxies remains under discussion in the literature. Evidence from concordance studies in cancer suggests that this may depend on the domain being assessed. For example, proxy–patient concordance in cancer for emotional functioning was better with the proxy–patient perspective [30], as was reporting of the symptom of diarrhoea [31]. In contrast, concordance was better for the proxy–proxy perspective for cognitive and role functioning [31]. In dementia, findings from a recent review [32] and concordance study [33] suggest that the proxy–patient perspective may yield better concordance. Further complicating this issue is the dearth of research on whether and how proxies use a specific perspective in practice. At the time of writing, there is no evidence on the extent to which proxies adhere to any perspective when completing measures [34]. Understanding how proxies use perspectives is important for informing the design and evaluation of proxy-reported measures, including those that evaluate HRQoL.

The EQ-5D is an HRQoL measure that is commonly used but has had suboptimal performance in conditions such as dementia [35] that may require proxies. A new generic measure, the EQ-Health and Wellbeing (EQ-HWB) instrument was developed; EQ-HWB development included input from carers [35]. The EQ-HWB has a version for proxy reporting [36]. Notably, the EQ-HWB’s 7-day recall period was described as “a pragmatic decision,” and the potential for reconsidering it based on performance evidence was stated [35]. Our study therefore sought to provide evidence on this new measure, whilst contributing to a broader area (recall periods and perspective-taking in proxy reporting) for which additional evidence is needed.

The specific aims of this paper were to explore: (1) how carers interpret the recall period in the proxy version of a new generic PROM, the EQ-HWB-9 (previously named EQ-HWB-S [37]), when reporting on the health and well-being of persons with dementia (Objective 1); and (2) which perspective(s) proxies use when completing the measure (Objective 2). For contextualisation and clarity, we also sought to assess the extent of participants’ prior experiences of reporting on the health and well-being of the person with dementia.

## Methods

### Study design

A qualitative, descriptive design was used to gain an in-depth understanding of how carers interpret the recall period and items of the proxy version of the EQ-HWB-9 [38]. The study was conducted between June and September 2024 and was approved by the University of Tasmania Human Research Ethics Committee (Project H0030051). Informed consent was obtained from all study participants. Findings on participants’ perceptions of the EQ-HWB-9 items, which were also explored in this study, will be reported separately. Reporting adheres to the COnsolidated criteria for REporting Qualitative research (CORE-Q) [39] (Online Appendix A).

### Participants

Individuals with experience as paid or unpaid carers were recruited using convenience sampling through a webpage linked to the Understanding Dementia Massive Open Online Course (UDMOOC), which is run by the University of Tasmania and has had previous enrolments of > 20,000, most of whom live in Australia [40]. UDMOOC participants have previously been involved in the development and validation of a dementia literacy tool, the Consumer Access Appraisal and Application of Services and Information for Dementia (CAAASI-Dem) [41]. This recruitment approach meant that the number of people who may have seen the advertisement but did not participate could not be recorded.

Individuals were eligible to participate if they were at least 18 years of age, living in Australia, currently providing care (paid or unpaid) for someone living with dementia, and had previously reported on behalf of the person with dementia about their health and well-being. The eligibility criterion of having previously reported on behalf of someone was included to ensure participants had experience as proxies. After reviewing the online information sheet and consent form, participants indicated their consent to participate by providing their contact information via secure REDCap (Research Electronic Data Capture) database [42, 43] hosted at the University of Tasmania for interview scheduling. A research assistant re-confirmed consent when scheduling the interviews.

### Data collection

Semi-structured interviews were conducted online using video-conference (Zoom© [44]). If participants indicated they did not have access to a webcam for the interview, one was provided to them prior to the interview. Participants also received a hard copy of the proxy version of the experimental EQ-HWB-9 (2022) questionnaire (English for Australia; version 1) in advance to mitigate against any possible technical difficulties with screen sharing. All interviews were conducted by the same interviewer (CN), a female health economist (PhD) with experience in qualitative research on PROMs. The interviewer had no prior or continuing relationships with any of the participants. Participants knew that the researchers were not involved in their care and the purpose of the research. Participants were advised to be alone with the researcher during the interviews. In a few cases, the person with dementia occasionally entered the room and carers briefly attended to them before returning to continue the interview alone. Such interruptions were minimal and, from the interviewer’s perspective, did not affect the quality or completeness of the data collected.

At the beginning of the interview, carers were asked to provide demographic information, including gender, age, and highest level of education. They were also asked about their relationship to the person with dementia, whether they identified as a primary, joint or secondary carer, how many days they saw the person with dementia, and their living arrangement with the person with dementia. Carers were then asked what being a primary/secondary/joint carer for the person with dementia meant for them. To understand their prior experience of proxy reporting, carers were asked “Have you ever provided a report about the health and well-being of the person you care for?” and prompted to expand on what circumstances or situations, and how often they provide such a report.

Carers were presented with the proxy version of the EQ-HWB-9, which begins with the following instruction: “For each question below, please select the one response that you think best describes the person in the last 7 days”. The EQ-HWB-9 includes nine items measuring mobility, daily activities, exhaustion, loneliness, cognition, anxiety, sadness/depression, control and physical pain with five response options [35]. In addition, three positively-worded items from the longer EQ-HWB version—feeling accepted by others, feeling good about themselves, and doing the things they wanted to do—were also presented [35]. These were included to explore the impact of phrasing on proxy report. Previous work examining phrasing for experience measures suggests some slight differences for proxy report [45], however information on this in the proxy-reported outcome context is limited. This was an exploratory objective.

Carers were asked to think aloud as they went through the questionnaire and were prompted as needed, following the topic guide developed for this study (Appendix B). Prompts to explore participants’ use of the recall period included: “What particular things were you thinking of to help you answer?” and “What do you think about these options over ‘the last 7 days’?” As health fluctuations are common in the context of dementia and may affect proxy recall [46], to examine any variation in the constructs assessed by the EQ-HWB-9, carers were prompted, “Was there variation during the last 7 days?” for each item. Proxy perspective was explored after completing the questionnaire items by asking: “Thinking about the questionnaire as a whole, did you answer the questions based on what you observe, or what you imagine (the person you care for) would say?”. Finally, participants were asked about any aspects of the questionnaire they found difficult.

Interviews were audio-recorded and transcribed verbatim, consistent with best practice recommendations [47, 48]. Participants were given the option to receive and review their transcripts for corrections and comments. The interviewer made field notes on observations during and after the interviews. A saturation grid documented the consistency of concepts and emergence of new themes, and recruitment continued until ‘information power’ was reached (i.e., no new information emerged) [49]. No repeat interviews were carried out. Transcripts were de-identified prior to analysis, and data were imported into NVivo v14 software for management and analysis.

### Analysis

We used an integrated thematic analysis involving inductive coding to capture new insights shared by participants and deductive coding as guided by the EQ-HWB-9 items [50]. Transcripts were initially coded inductively line by line to ensure that insights were captured irrespective of their fit to established theory or the specific EQ-HWB-9 item to which they referred. Codes were subsequently organised into categories and sub-categories. The next step of analysis was more deductive, involving classification of the codes according to the relevant EQ-HWB-9 item and noting when multiple participants reported similar experiences or interpretations of an item.

The first five transcripts were independently coded by two researchers (JR and CN), and then reviewed together to develop an initial analysis framework. CN then coded the remaining interviews, with the coding framework regularly discussed with JR, TL and BM and refined where necessary. The final framework was applied to all transcripts. Background characteristics were compared against emerging categories to identify whether specific perspectives or challenges were more commonly expressed by subgroups.

The summary and interpretation of the findings were reviewed and confirmed with the study advisor (MB), who has lived experience of caring for someone living with dementia. Participants were also invited to review a one-page summary of the overall study’s findings and disagree or suggest refinements if necessary. Quotes from primary, secondary and joint carers are denoted by the letters P, S and J, respectively.

## Results

### Participant recruitment and contextual information about participants

**Fig. 1:**
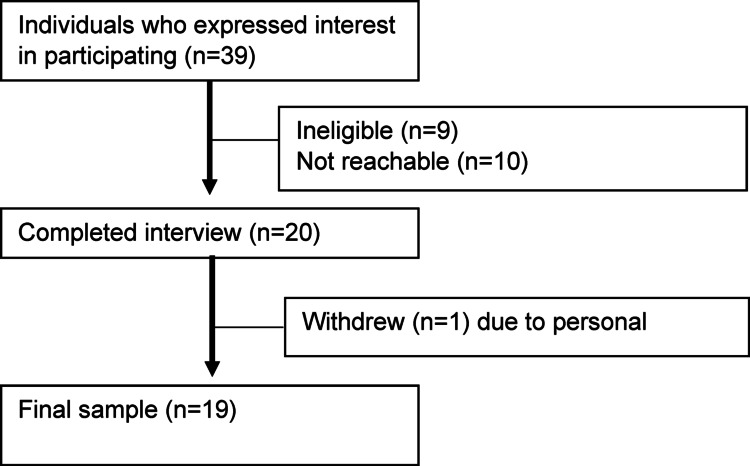
Recruitment flowchart

Of the 39 potential participants who were contacted, 19 completed interviews (Fig. 1). Table 1 presents the characteristics of the participants. Interviews lasted between 49 and 69 min (mean of 60 min). Appendix C presents the saturation grid and outlines the identified categories and subcategories. No new concepts or themes emerged following the tenth interview.

**Table 1:** Characteristics of participants (n = 19)

	Participants (n)
Gender	
Male	2
Female	17
Age	
45–54	6
55–64	6
65–74	3
75–84	3
85 and above	1
Highest level of education	
Secondary school	1
Vocational training	5
Undergraduate degree	6
Postgraduate degree	7
Relationship to person with dementia	
Daughter/son	9
Partner/spouse	3
Friend	3
Non-immediate family member	2
Former spouse	2
Whether identified as primary carer	
Yes	11
No, joint carer	6
No, secondary carer	2
How many days each week they saw the person	
1–2	4
3–4	4
5–6	2
7	9
Living situation of care recipient	
Lives with carer	7
In residential care	2
Other living situation	10

When asked what being a carer meant to them, most described it in terms of being responsible for providing support with daily activities and spending time with the person with dementia. Nearly half of the primary carers also reported having to “supervise” (P12) or “liaise” (P3) with paid support workers for tasks such as cleaning and showering assistance. Some primary carers also described being responsible for major decisions on the behalf of the person with dementia, including financial matters and holding power of attorney.

The weight, mostly, is about the fact that I will be responsible for making decisions about her health and wellbeing. And that's huge. (P10)

Almost all joint carers described their role as sharing responsibilities by “taking turns” (J7) or allowing the other carer(s) “take a break” (J18). While the frequency with which they saw the person with dementia did not appear to determine whether someone identified as a primary carer, the level of involvement in day-to-day life emerged as a key factor in how participants perceived their caregiving role.

I was his primary carer until he went into care… I go in (the care facility) four days a week to keep him in a routine, keep him settled and make sure that he is being cared for how I want him cared for. (S11)

### Contextualisation: prior experience of proxy reporting

Fourteen carers described having provided a report on behalf of the person with dementia regarding their health and wellbeing. This was typically done during joint attendance at medical appointments. Several carers described the person needing “prompting now and again” (P3) while being mindful of “including (the person) in the question-asking” (P9) and “getting (them) to speak as much as (they) can” (P2). A few carers also reported keeping a record to monitor signs of deterioration.

In other instances, some participants provided regular updates to family members, other carers, aged care facilities and other service providers. These reports were sometimes part of “onboarding into different service programs” (P14), or were required for entering residential care, which could involve “questions about (their) mobility, care needs, eating, and comprehension” (J5).

### Use and interpretation of the recall period in retrieval (objective 1)

#### Variations in aspects of well-being

To examine any changes in the constructs assessed by the EQ-HWB-9, carers were asked, “Was there variation during the last 7 days?” for each item. For most items, carers recalled fluctuations in the person with dementia’s behaviour within the last 7 days, which were influenced by various settings, such as being inside or outside, the time of day, and the specific activity the person with dementia was engaged in.

If it’s first thing in the morning and we go for a walk, he’s fine, but if we go shopping, quite often times he needs to lean against the shopping trolley, or I’ll have him by the arm and he gets really, really tired (P19)

However, other carers did not consider such fluctuations as variations. Instead, they interpreted the question as referring to whether there had been any changes from the person with dementia’s “new normal” (S11). This discrepancy in interpretation appeared more pronounced for the EQ-HWB-9 questions relating to difficulty (Q1, getting around and Q2, day-to-day activities), compared to the other items.

Her level of getting tired is the way she always was. Not always – as in post-surgery – it’s the same. (J7, about Q2)

I'm constantly monitoring and scanning, so when I look at the ‘last seven days’ it's almost like, “When's the last time I saw her having difficulty with something?”, and is that for me a progressive change or is that a sudden change in her capacity? (P14)

Drivers contributing to these types of shifts were typically changes to routine, such as starting new treatments, lifestyle changes, illnesses, falls, hospitalisation, and progressive deterioration associated with dementia.

Carers’ retrieval strategies in relation to their use of the recall period were categorised into two main approaches, as detailed below.

#### Strategy 1: extending the recall period to capture significant events

Most carers found it easy to answer items where observations could be made (Q1, getting around; Q2, day-to-day activities; Q5, concentrating) or were more readily expressed through verbal communication by the person with dementia (Q3, exhausted; Q9, pain) within the last 7 days.

She often says ‘I have no energy, I feel really exhausted’ so she verbalises that. (P13).

There did not appear to be any association between adherence to the recall period and how often carers saw the person with dementia. Carers who did not see the person with dementia as regularly also referenced times they spoke with the person over the phone or interactions with other carers.

Some carers referred to significant events or the person with dementia’s self-report that occurred outside of the recall period, believing these were important to consider when answering.

The last seven days, I'd say maybe some difficulty, whereas if I go back nine days for that I'd say a lot of difficulty… I think that the tolerance for seven days is eight days to ten days. (P14)

These earlier events were considered alongside their observations, or the lack thereof, from the past 7 days. In some cases where carers could not recall relevant information from the past 7 days, their responses were informed solely by events outside the recall period.

He does share these feelings and thoughts (about feeling accepted) with me, (but) the last seven days we didn’t have much of an opportunity to talk about this… I would still assume that he feels that way. (S1)

It’s hard to tell (if mum felt lonely), but if it was the “last 14 days”, I can tell you a very interesting occurrence. (P3)

#### Strategy 2: drawing on the past to contextualise recent behaviours

All carers compared behaviours observed within the past 7 days to earlier behaviours, considering whether these had changed or were still affected, to help inform their responses.

Six months ago, I would have said slight difficulties, now I would say some difficulties… Her gait was different six months ago. (P13)

Carers were more likely to draw on such comparisons for items related to feelings, including Q4 (lonely), Q8 (no control), and the three positively-worded questions (Q10, accepted by others; Q11, felt good about themselves; Q12, could do the things they wanted), especially when the person with dementia had not clearly expressed these feelings in the past 7 days. In these cases, carers reflected on earlier events where the person with dementia expressed similar emotions, such as changes in living arrangements and social situations, to guide their responses.

I can compare it to before, when she was admitted to hospital. If I came over with my husband and did what we've been doing this past week – like cleaning the house, getting things in order, doing her laundry – she wouldn't have wanted us to do it really, she would have been up and round trying to stop us, but she hasn't at all this past week. That's been a marked difference. (P10)

Carers also referred to the person with dementia’s personality traits, idiosyncrasies, and mannerisms prior to their diagnosis of dementia when discussing all EQ-HWB-9 items, though this was rarely done for Q1 (getting around), Q2 (day-to-day activities), Q3 (exhausted), and Q9 (pain). Some behaviours were subtle and likely only recognisable to those with an intimate understanding of the person with dementia.

He just broke into song and started singing, and that’s the kind of thing he does. When he’s feeling anxious… he’d just break into song. That was his way of deflecting what was happening. (J18)

He doesn't actually say he’s lonely. He'll say it in a different way, that he's wanting to fix his flaws, and he's wanting to get back into shape. (J17)

For Q6 (anxious) and Q7 (depressed), most carers also referenced the person with dementia’s history of anxiety and/or depression, prior to dementia diagnosis, to inform their responses.

#### Application of recall in response strategies

Carers used different strategies to answer the items assessing difficulty (Q1, getting around; 2), including averaging behaviours, focusing on the most challenging moments, or considering how frequently certain behaviours were observed—within the context of their observations and any fluctuations, typically over the past 7 days.

Some days it’s okay, he might get three or four of the things down – like the knives and forks and serviettes, but forget the salt and pepper. But the last two days, almost every implement has to be instructed…. I’d still say it’s a lot of difficulty (P12)

For the pain severity question (Q9), carers gave examples of how they linked each response option to specific behaviours that occurred within the recall period. Although frequency of pain was sometimes mentioned, this did not appear to significantly influence carers’ responses.

My mum is very stoic, so the fact that she is saying something about the pain indicates that it's there. I think if it was severe we would be hearing about it every day, but we're not… I think it qualifies as moderate because it's interrupting her sleep. (P10)

For the remaining questions assessing frequency, most carers interpreted response options in terms of how many days a behaviour occurred in the past 7 days, while some considered the number of times the behaviour occurred in the past 7 days instead. For Q5 (trouble concentrating or thinking clearly), some carers also factored in how often they needed to assist the person with dementia.

### Proxy perspective and its implications (objective 2)

All carers reported answering the EQ-HWB-9 questions based on their own observations and perceptions of the person with dementia (proxy–proxy perspective). Indeed, carers found it challenging to answer items about constructs that were perceived as less observable or expressed. When asked to report on the person with dementia’s feelings in the last 7 days, carers relied on behaviours that occurred within this period that could serve as indirect indicators. A notable example was carers observing how the person with dementia engaged with others in a social setting, or whether they had “had plenty of company” (J20), to assess feelings of loneliness (Q4) and acceptance (Q10).

She wouldn't exactly tell you, ‘I feel like I'm able to be myself. I feel belonged’. How do I know whether she feels accepted other than by demonstrating that she is participating and not withdrawing from activities? I have no other measure to know how she feels. (J5)

However, some carers acknowledged that “you can be sitting with someone and chatting away, and you could still feel lonely” (P2). Carers also described using a range of other observations for Q4 (loneliness), such as the person with dementia verbally expressing a desire for interaction, not wanting the carer to leave, or talking about missing someone. For Q10 (felt accepted by others), most carers also based their responses on how much other people appeared to be accepting of the person with dementia.

I’d like to say often (she felt accepted), but I’m going to say sometimes… I felt the ladies in the club, some of them might have treated Mum differently because she suffered this condition… they talk about Mum like she’s no longer here. (P3)

Most carers also relied on various indirect indicators to assess feelings of control over day-to-day life (Q8), such as whether the person with dementia could remember “what we’re going to be doing each day” (P19), the level of resistance encountered during caregiving tasks, and the extent of autonomy carers were able to provide.

I think there are times when he feels I’m a little bit bossy. He hasn’t got perhaps as much control as he would like. But then if he says to me, ‘Look, I can do this myself,’ and I say, ‘Fine, go ahead,’ then he has had some control over his life, hasn’t he, because he explained to me that he wants to do it himself and does. (P9)

In addition to the proxy–proxy perspective, nearly half of the carers also imagined how the person with dementia might answer if asked directly (proxy–patient perspective). No carer solely took the perspective of the person with dementia (proxy–patient perspective), with some expressing concern that reduced insight could impact responses.

It would be hard for him to make sense of some of these questions and know how and what that means for him. (J17)

In some cases, the person with dementia had been asked a similar question or expressed their feelings within the past 7 days, but carers disagreed with their self-assessment. This inconsistency was most notable for questions about pain (Q9) and feelings of control (Q8).

I don’t know whether she feels that she has no control over her life. I don’t think she does feel this because she still thinks she can do everything; she’s told me ‘I can go on a holiday’ and I said, ‘well who would look after you?’ ‘Oh, I can look after myself.’ (P13)

Although not a primary factor, some carers also considered how the pre-dementia individual might perceive themselves now, particularly when responding to Q7 (sad/depressed) and Q11 (felt good about themselves) and Q12 (could do the things they wanted).

She fought the disease for such a long time, and that’s why she did bridge and online computer games, memory games. So, I don’t think she’s depressed, but I think she’s sad that she lost the fight. (P3)

## Discussion

This study explored how carers used the recall period in the proxy version of the EQ-HWB-9 when reporting on the health and well-being of person with dementia, and the perspective(s) they used. Most carers were able to recall relevant observations of the person with dementia within the past 7 days across the EQ-HWB-9 domains. However, adherence varied depending on whether key events or self-report occurred slightly outside the recall period and the extent to which carers drew on the person with dementia’s past behaviours and personalities to interpret recent ones. Previous studies have similarly shown that carers tend to compare the person with dementia’s current health to their pre-deterioration state [51]. Carers used both proxy–proxy and proxy–patient perspectives.

Capturing changes in health and well-being using questionnaires with a specified recall period has been shown to be challenging for both patients and proxies [46, 52–54]. For PROMs, evidence suggests that shorter recall periods (e.g., today) may be insufficient [53] to support adherence to the recall period amid health fluctuations. Individual preferences for recall periods for PROMs can vary based on the domain being assessed [55]. However, for proxies, longer recall periods may be helpful for capturing health fluctuations, with some proxies suggesting periods of 2–4 weeks in earlier work with the EQ-HWB [36]. In the context of dementia, where health fluctuations are common, proxies are likely to adhere less to the EQ-5D’s recall period when the person with dementia experiences more frequent health fluctuations [46]. In any case, for measures (both proxy- and patient-reported) more generally there is a clear need to understand how best to improve recall period adherence and recall consistency, and how variability in recall period use may impact measure validity [56]. When proxy-reported measures are used, it may be worth evaluating the extent of adherence to the recall period.

When carers are asked to report as proxies, they are often instructed to report from a specific perspective [36] or perspectives [57], and provision of such instructions is recommended by the International Society for Quality of Life Research (ISOQOL) proxy task force recommendations [29]. However, evidence on whether proxies adhere to the specified perspective is often lacking and the limited available evidence suggest adherence can be inconsistent [36]. Our findings point to the need for a better understanding of how carers perceive and use proxy perspectives when answering as proxies. Rather than focusing on adherence to a specific perspective, one alternative is to ask proxies to answer from both perspectives, as is done in the ASCOT-Proxy [57]. In dementia, proxy reports using the proxy–patient perspective appear to have greater agreement with patient self-report compared to the proxy–proxy perspective [32]. Similar findings were reported in a review of studies from residential care homes [28]. However, concordance is not the sole relevant metric for evaluating the performance of proxy reports, particularly in situations where self-report is not available, such as in cases of severe dementia. Collecting reports from both perspectives may provide useful, complementary information [26] and be more acceptable for proxies [57]. While our study supports the feasibility of this approach, further research is needed to better understand how proxies interpret and apply instructions on perspective when completing questionnaires, including questionnaires assessing HRQoL, and how adherence to these perspectives might be improved. Additionally, a dual perspective approach may be challenging when applying measures in the context of health technology assessment or regulatory evaluation, where a single approach may be preferable. Going forward, studies with single perspectives may wish to evaluate adherence to the perspective, and developers of future measures should consider partnerships with carers as part of measure development and thorough qualitative assessment of measure phrasing.

Lastly, this study was one of few that gathered contextual information about proxies who reported about someone else’s health, which is important for advancing our understanding of how proxy–patient relationship characteristics influence proxy reporting [58–60]. In dementia concordance studies, most proxies have been residential aged care staff [28], as was the case in a recent assessment of the EQ-HWB for residential aged care [36]. In this study, we found that community-based proxies had experience of providing updates to family members, other carers, and service providers. As proxies will be unavoidable in some circumstances, including in dementia, there have been recommendations to improve proxy reporting [61, 62]. Understanding more about the experience of proxy reporting may be a useful first step in this regard.

### Limitations

This study had several limitations. First, participants were recruited via convenience sampling through a webpage linked to an online course, which may have limited the sample to individuals with a personal interest in the topic of dementia and/or higher levels of digital literacy. The study was also limited to individuals who lived in Australia and spoke English, and the findings may not generalise to other populations and patient groups where proxy reporting is required. Insights into participants’ cognitive processes were additionally limited to what they verbalised during think-aloud and probing, which varied between individuals. In proxy reporting for a person with dementia, emotional factors may have led to the omission of sensitive topics. Additionally, we did not ascertain how easy carers found it to remember the person with dementia’s ability to self-report, which may be relevant for the choice of proxy perspective. Although we had a broad representation of carers with varying levels of involvement, future research could explore in greater depth how different proxy–patient subgroups (e.g., relationship type, carer role, cultural background, degree of prior experience with proxy reporting) may influence findings. Finally, in this study nearly all participating carers were female. It is not clear if this is a limitation. Women have been the majority of proxy participants in other studies involving proxies in dementia [63–65] and cancer [66], suggesting that this is not unique to our study.

## Conclusions

Findings from this qualitative study involving carers of persons with dementia suggest that while the 7-day recall period for the EQ-HWB-9 was generally followed, adherence varied based on several factors. These included whether relevant observations occurred just outside the recall period and the extent of reliance on the person with dementia’s past as a reference point. Many carers also used both a proxy–proxy and proxy–patient perspective, with conflicts between the two noted, highlighting the need for further considerations regarding proxy perspective instruction and its adherence. The inclusion of such instruction and item wording changes may thus be needed to improve recall consistency and applicability of the proxy version of EQ-HWB-9 in the context of dementia.

## Supplementary Information

Below is the link to the electronic supplementary material.

Supplementary Material 1

Supplementary Material 2

## Data Availability

The data used for this study cannot be shared due to privacy/confidentiality concerns.
