# Supplementary material for: Carers’ interpretation of the recall period and perspective-taking when completing the EQ health and wellbeing instrument (EQ-HWB)-9 as proxies for people with dementia: a think-aloud study
Source: Qual Life Res. 2026 Apr 1;35(5):115. doi: 10.1007/s11136-026-04230-y (PMC13043530; doi:10.1007/s11136-026-04230-y)
Supplement: Supplementary file 1 — Supplementary Material 1 [file 11136_2026_4230_MOESM1_ESM.pdf]

COREQ (COnsolidated criteria for REporting Qualitative research) Checklist

A checklist of items that should be included in reports of qualitative research. You must report the page number in your manuscript where you consider each of the items listed in this checklist. If you have not included this information, either revise your manuscript accordingly before submitting or note N/A.

Topic	Item No.	Guide Questions/Description	Reported on Page No.
**Domain 1: Research team and reflexivity**			
Personal characteristics			
Interviewer/facilitator	1	Which author/s conducted the interview or focus group?	6
Credentials	2	What were the researcher's credentials? E.g. PhD, MD	6
Occupation	3	What was their occupation at the time of the study?	6
Gender	4	Was the researcher male or female?	6
Experience and training	5	What experience or training did the researcher have?	6
Relationship with participants			
Relationship established	6	Was a relationship established prior to study commencement?	6
Participant knowledge of the interviewer	7	What did the participants know about the researcher? e.g. personal goals, reasons for doing the research	6
Interviewer characteristics	8	What characteristics were reported about the inter viewer/facilitator? e.g. Bias, assumptions, reasons and interests in the research topic	6
**Domain 2: Study design**			
Theoretical framework			
Methodological orientation and Theory	9	What methodological orientation was stated to underpin the study? e.g. grounded theory, discourse analysis, ethnography, phenomenology, content analysis	7
Participant selection			
Sampling	10	How were participants selected? e.g. purposive, convenience, consecutive, snowball	6
Method of approach	11	How were participants approached? e.g. face-to-face, telephone, mail, email	6
Sample size	12	How many participants were in the study?	8
Non-participation	13	How many people refused to participate or dropped out? Reasons?	8, Figure 1
Setting			
Setting of data collection	14	Where was the data collected? e.g. home, clinic, workplace	6
Presence of non-participants	15	Was anyone else present besides the participants and researchers?	6
Description of sample	16	What are the important characteristics of the sample? e.g. demographic data, date	6, 8, Table 1
Data collection			
Interview guide	17	Were questions, prompts, guides provided by the authors? Was it pilot tested?	6,7, Appendix
Repeat interviews	18	Were repeat inter views carried out? If yes, how many?	7
Audio/visual recording	19	Did the research use audio or visual recording to collect the data?	7
Field notes	20	Were field notes made during and/or after the inter view or focus group?	7
Duration	21	What was the duration of the inter views or focus group?	8
Data saturation	22	Was data saturation discussed?	7
Transcripts returned	23	Were transcripts returned to participants for comment and/or	7

Topic	Item No.	Guide Questions/Description	Reported on Page No.
		correction?	
**Domain 3: analysis and findings**			
Data analysis			
Number of data coders	24	How many data coders coded the data?	8
Description of the coding tree	25	Did authors provide a description of the coding tree?	6,7, Appendix
Derivation of themes	26	Were themes identified in advance or derived from the data?	7
Software	27	What software, if applicable, was used to manage the data?	7
Participant checking	28	Did participants provide feedback on the findings?	7,8
Reporting			
Quotations presented	29	Were participant quotations presented to illustrate the themes/findings? Was each quotation identified? e.g. participant number	9-14, Appendix
Data and findings consistent	30	Was there consistency between the data presented and the findings?	9-14
Clarity of major themes	31	Were major themes clearly presented in the findings?	10-14
Clarity of minor themes	32	Is there a description of diverse cases or discussion of minor themes?	10-14

Developed from: Tong A, Sainsbury P, Craig J. Consolidated criteria for reporting qualitative research (COREQ): a 32-item checklist for interviews and focus groups. *International Journal for Quality in Health Care*. 2007. Volume 19, Number 6: pp. 349 – 357

Once you have completed this checklist, please save a copy and upload it as part of your submission. DO NOT include this checklist as part of the main manuscript document. It must be uploaded as a separate file.

Appendix B: Topic guide

Probes and structure based on other topic guides, as gratefully acknowledged in the footnote[1].

Interviewer to have:

- Consent form
- Participant Information Sheet
- EQ-HWB proxy version for Australia (version 1)

1. **Welcome**

Researcher introduces themselves
Researcher welcomes participants, begins with icebreaker comments

2. **Introduction and taking consent for recording**

Thank you for agreeing to be part of this study.

Researchers at the Menzies Institute for Medical Research at the University of Tasmania, the University of Technology, Sydney in NSW, Curtin University in WA and at the University of Sheffield in the UK are trying to understand more about a new questionnaire that measures health and how people feel and function on a personal and social level, and evaluate their lives as a whole[2]; this is sometimes called well-being.

In this study, we want to understand more about a new questionnaire measuring health and well-being. We hope to be able to measure the health and well-being of people living with dementia more accurately. This questionnaire asks about the difficulties people may have with activities, people's mood and emotions, positive feelings, pain, and physical discomfort. It has 25 questions in total.

The reason we are talking to people who care for people living with dementia is that sometimes the person living with dementia might not be able to answer for themselves. In these situations, carers can be asked to talk about another person's health and well-being. In this study, we would like to know more about how people, such as friends or relatives, looking after people with dementia answer questions about the quality of life of the person living with dementia.

We will begin by asking you a few questions about yourself and then we will ask you questions about the health and well-being of the person with dementia whom you are caring for. There are

---

[1] Carlton J, et al., Generation, Selection and Face Validation of Items for a New Generic Measure of Quality of Life: The EQ-HWB. *Value Health* 2022; 25(4): 512-524, doi: 10.1016/j.jval.2021.12.007; Patrick DL, et al., Content Validity—Establishing and Reporting the Evidence in Newly Developed Patient-Reported Outcomes (PRO) Instruments for Medical Product Evaluation: ISPOR PRO Good Research Practices Task Force Report: Part 2—Assessing Respondent Understanding. *Value Health* 2011; 14: 978 – 988. doi: 10.1016/j.jval.2011.06.013; Brod ML, Tesler LE, Christensen TL, Qualitative Research and Content Validity: Developing Best Practices Based on Science and Experience. *Qual Life Res* 2009; 18(9): 1263 – 1278. Doi: 10.1007/s11136-009-9540-9; Rand S et al, Developing a proxy version of the Adult social care outcome toolkit (ASCOT). *Health Qual Life Outcomes* 2017; 15:108. doi: 10.1186/s12955-017-0682-0; Pattinson R et al, Evidence of the content validity, acceptability, and feasibility of a new Patient-Reported Impact of Dermatological Diseases measure. *Front Med* 2023; 10:1020523. doi: 10.3389/fmed.2023.1020523

[2] The quote is: One of the most widely cited definitions of wellbeing is as follows: "wellbeing can be understood as how people feel and how they function both on a personal and social level, and how they evaluate their lives as a whole," Jarden A & Roache A, What Is Wellbeing? *Int J Environ Res Public Health* 2023; 20(6): 5006. doi: 10.3390/ijerph20065006

no right or wrong answers. It would be the best guess about the person living with dementia. This should take about an hour.

Do you have any questions? You can ask me questions at any time if you are unsure of anything.

You can stop this interview and withdraw at any time, you don't have to give a reason. If you do stop, this won't affect your relationship with any of the universities or any clinical care.

I will be recording this. Is this OK with you? We might use some of what you say as a quote, but you will not be identified. Is this OK with you?

3. **Questions about the carer**

To begin, I want to ask a few questions about you, such as how old you are, how long you were in education, and your experience of caring for someone living with dementia. This can help us understand if there are some differences in people's views about some parts of the questionnaire. If you don't want to give an answer to a question, for whatever reason, that is OK, and that does not mean we can't continue with the interview.

When we describe the characteristics of the people we interview for this study, we will only provide this information as a summary. You will be anonymous and not identified in any way from that information.

I am going to take notes on this section and won't start recording yet. If you prefer not to answer any question, please let me know.

- What is your age? Please tell me your age in years.

- How would you describe your gender?
    - Man
    - Woman
    - Non-binary
    - Other (specify)
    - Prefer not to say

- What is the highest level of study that you completed?
    - Primary school
    - Secondary school
    - University – undergraduate
    - University – postgraduate
    - Vocational training/TAFE
    - Prefer not to say

- What is your relationship with the [cared for person]?
    - Partner/spouse
    - Child
    - Other

- Paid carer
- Other (specify)

- Would you describe yourself as the primary carer of the [cared for person]?

- Do they live with you?
  - Yes
  - No – in residential care
  - No – other living situation

- How many days each week do you see the [cared for person]?
  - 7
  - 6
  - 5
  - 4
  - 3
  - 2
  - 1

I am now going to start recording.

- Earlier you answered you were/were not the primary/secondary carer of the [cared for person]. What does that mean for you?

- Have you ever provided a report about the health and well-being of the person you care for?
  - What were the circumstances or situations?
  - How often do you provide such a report?

4. Probing questions for EQ-HWB topic areas

Note for interviewer: not all probes need to be used

Thank you. I'm going to move to asking you about the person with dementia.

I am sending you the link to the questionnaire in the chat section. Please share your screen with me once it has loaded. If it's easier, you can look at the paper copy we sent you.

For this section, we don't need to know how you would answer if you were filling out the questionnaire on behalf of the person you are caring for. Instead, we are interested in what you think about the questions. We are interested in your views on the questions in general, and how and what you think about the time frame when you try to answer the question.

If at any time you feel tired or you'd like to take a break, we are happy to continue later or reschedule. Please just let me know.

<Recall Period> Let's start by looking at the time frame for this questionnaire.

- What does 'the last 7 days' mean to you when you think about the [name of cared-for person]?
- Can you talk me through how you would think about this time frame?
- Was this pattern/timing of [symptoms/experience] usual for [name]?

Let's have a look at this first set of questions. We'll start with question number 1. I will show you the questions and read out the questions, <Read out the question, starting with question 1>

[Probes for each item – not all of these have to be asked]

What particular things were you thinking of to help you answer?

What do you think about these options over 'the last 7 days'?

Do you feel you can describe this over the past 7 days?

- <if no>: What would you do if you felt you could not describe this over the past 7 days?

Was there variation during the last 7 days?

Were you thinking about particular things that happened in the past 7 days?

[Additional probes for questions 3 – 9. These does not have to be asked for every item in the set, nor do both probes have to be used if there is discussion of the response choices and the recall period.]

Would you be able to answer this question using these response choices – you don't need to tell me what the answer would be, just if you think you could answer.

How do you think about these response choices over 'the last 7 days'? I'm interested in knowing if you think they are easy or hard to choose.

[Additional probes for questions 10 – 12, the positively worded questions. These does not have to be asked for every item in the set, nor do both probes have to be used.]
What do you think about this question?

Do you think there is a better way we could ask about this?

5. Wrap-up

Now I have a few last questions for you after you have looked at the whole questionnaire and talked about it with me.

Thinking about the questionnaire as a whole, did you answer the questions based on what you observe, or what you imagine (the person you care for) would say?
Were there any parts of the questionnaire that were harder than others? If so, which parts?

Thank you very much for talking with me today. Your contribution is very important and it is helpful to the research team to get your input.

Do you have any questions you would like to ask?

Is there anything else you would like to say about the questionnaire?

Would you like to see a transcript of this interview?
[If yes: Talk about secure email sharing]

Would you like to receive a summary of the results of the study, once the study has finished?
[If yes: Say that we will retain their contact information for this purpose, and confirm that results can be emailed. If not, ask for preferred form of results return]

Appendix C: Illustrative participant quotes for themes and subthemes

**Category** (Subcategory and/or EQ-HWB-9 item)	**Illustrative participant quotes**
**Prior experience of proxy reporting**	
Prompt person living with dementia (PLWD) during appointments	"*Any time he goes to any doctor appointments, I always go in with him. If there's questions that the doctor's asking, often times [NAME]'s vocabulary just goes blank on things, so I'll provide words for him and then he'll say as much as he can and then I'll fill in gaps if there's things that are still needed or lacking.*" (P19)<br><br>"*The doctors ask him how he is and he doesn't remember why he's there for or anything. I assist him with all that. I get him to speak as much as he can. Sometimes, I might just have to prompt him, and then he can speak for himself.*" (P2)<br><br>"*She prefers it when the medical carers, or the professionals, or whoever we're dealing with, deal with her rather than deal with me – it's like the tripartite, she feels left out of the conversation if they're only talking to me. I make sure, whoever we're dealing with, if it's to do with her it's presented to her so that I'm the third party, not Mum.*" (P3)
Provide updates to family/care providers/services	"*I have to do a survey, every month in regards to his care, to let them know how I believe they're going but how he's going as well. They being the doctors and stuff. I do all that for him. All his medical stuff, that all is done through me.*" (S11)<br><br>"*The assessor came to the house. That was an assessment for her own package funding level to increase… I've done that twice now with the [ASSESSMENT NAME] assessment team. At the moment – recently, in the last couple of weeks, I've been providing reports because I'm trying to get her into residential care. So, of course, they're asking me questions about her mobility, care needs, eating, comprehension.*" (J5)
**Variations in aspects of well-being**	
Q1 getting around inside and outside? (using, for example, a walking stick or wheelchair if they normally use them)	"*It fluctuates… It depends where we are. I find when we're out, like we were out for coffee with a group of people, I'm trying to keep him socialising, I find that his behaviour is a lot better when he's out than when he's home.*" (P12)<br><br>"*Since her diagnosis, she's continued to live independently, she's continued to actually do some work even from home, and she's continued to do a lot of her shopping, going to church. She's still lived quite an independent life. A lot of that has changed dramatically since the fall earlier this month.*" (P10)

	"*I think in the last seven days, except for one night where we needed to use the wheelchair because I couldn't get her to walk with the walker and that was one of those morphine nights which she was just totally off. But otherwise, I would think she's the same, using her things. So I would say no difficulty because basically she is supported with the walker and she manages, she knows she needs to release the brakes, she needs to hold on.*" (J7)
Q2. doing day-to-day activities? (e.g. working, shopping, housework)	"*It fluctuates. It's through the day that he'll do it as well. It's not even just over the week. It goes up and down where he's good sometimes and then other times, not. It's throughout the day. Part of that is his medication and part of that is just the dementia and how that goes. Part of it's tiredness. It's never the same. It's a constant up and down thing or a rollercoaster… Very rarely do I see him just be what I would consider normal or the new normal.*" (S11)<br><br>"*I'm constantly monitoring and scanning, so when I look at the last seven days it's almost like, 'What's the last time I saw her having difficulty with something?' And is that for me a progressive change or is that a sudden change in her capacity?*" (P14)<br><br>"*Her level of getting tired is the way she always was. Not always – as in post-surgery – it's the same.*" (J7)
Q3. felt exhausted?	"*Often in the morning she might say, 'I didn't sleep very well, I feel tired,' but by the afternoon she might say she's got a lot of energy. And vice versa as well.*" (P14)<br><br>"*No (fluctuation in exhaustion). You can nearly time it be clockwork. About 9:30 in the morning, closing his eyes, resting his eyes, as my nanna would always call it.*" (P2)<br><br>*"She's had a cold, so I think she has slept a bit. I don't actually know… I can only assume, but that's not really that good, because I haven't seen her. The last time I saw her was outside of those seven days. When she talks on the phone, she doesn't say that she feels exhausted. She says that she's having – She's good with just having rests and she'll go for a lie down if she needs one.*" (P8)<br><br>"*I don't know if it's exhaustion or just his age... He was always an early riser – get out and get the paper. He lost that.*" (J18)

Q4. felt lonely?	"*I think it has increased, really. He has been feeling really lonely after his son passed away, and that's gradually increasing. And it has probably increased in the last seven days*." (S1)<br><br>"*The day before yesterday, when I spoke to her on the phone, she sounded a little bit down. And I think she sounded a bit – maybe it's my own interpretation, but I think she sounded a little bit lonely. And that was in the evening. I think the evenings are the worst*." (J20)
Q5. had trouble concentrating or thinking clearly?	"*I think she was just better at masking (her cognitive declines) before the fall*." (P10)<br><br>"*Her ability to say clearly what she needs starts getting a little, I think, disturbed as the day progresses and the panic is starting to build, the anxiety is starting to build up, because it's starting to get dark and 'I don't know what's going to happen tomorrow, and where are all these people going to go?', and all that kind of thing. So it's all connected to that more than anything, I think, her thinking*." (J7)<br><br>"*Familiar tasks he's okay with, but if it's concentrating, like reading a book or something like that, and trying to understand what's going on, that's very, very difficult for him most of the time*." (P19)
Q6. felt anxious?	"*I would say throughou) the day there would definitely be that anxiousness that goes on for him because of his hallucinations. He will constantly say to me, 'I know that tonight's not going to be a good night. I know that this afternoon, there's going to be trouble.'… It is a constant thing that he says that makes me know that he's anxious. That used to not be the case when I would visit him*." (S11)<br><br>"*It fluctuates quite a lot. If I'm in meetings at work and I can't answer her calls, then – because often she's calling with a question which if it's not answered – depending on the day, depending on what it is*." (P14)
Q7. felt sad or depressed?	"*She's much worse in the evenings, she'll feel a bit sad or depressed. We did discuss this with her GP. At one period of time she was on antidepressants because, you know, just because you're elderly doesn't mean you don't get depressed. So that did help for a while*." (J20)
Q8. felt they had no control over their day-to-day life? *(e.g. had no choice to do things or have things done for them as*	"*I would put sometimes because it depends what happened the day before and what happens when I'm not there, if somebody upsets her*." (P4)<br><br>"*I think since he's been in (aged) care, he's lost that control… Even before that though, he had felt like he had lost control of, I suppose, him being independent and things like that once he came out of hospital, which is when he came*

they liked and when they wanted)	*home and was living with me, and I was caring for him full-time. Because they'd taken his license off him. They'd taken the car license which meant that he didn't have a truck license. He didn't have a bike license. He'd lost his shooter's license, things like that. Everything was removed from him and taken away from him. I think that's where he then felt like he had lost control of himself.*" (S11)
Q9. how much physical pain you think the person had	"*She's not feeling any pain when she's not doing anything, when she's immobile, but when she touches it, she'll feel pain. We say, 'Okay, well, don't touch it', but she'll forget. I think it qualifies as moderate because it's interrupting her sleep*." (P10)<br><br>"*He takes Osteomol three times a day, usually. Sometimes he only takes it twice. He takes two pills, so it could be anywhere from two to six pills he'll take in a day. If he didn't take those, I'd have to say it would be moderate.*" (P19)
Q10. felt accepted by others? (e.g. felt like they were able to be themselves and that they belonged)	"*I felt this morning that he didn't look as though he was included in the conversation, but that can happen, because he doesn't watch – or when he watches TV he doesn't take it all in, like the news or the football. So they may have been talking about something like that, and he didn't look as though he was processing any answers*." (P12)<br><br>"*That's a tough one because of that whole lonely thing that comes in the evening. If I go by the day and when we have the whole family together, as in the grandchildren and everybody in, she's in the middle of everything. She's laughing and talking to them, and asking them questions, and they are talking to her, and there is a whole part of family, like 'I belong and I'm all fine over there.' And then evening comes and its night, and she's all alone*." (J7)
Q11. felt good about themselves?	"*He does say that 'I was so good in this, but I lost interest in it now.' He has his own [POSSESSION]. I had my own [POSSESSION]. We used to brainstorm with each other, 'We're doing this, what to do,' and we picked each other's brain quite often. And he can't do simple mathematics now, or he doesn't know much about what's happening in the share market. And he says that, 'I was so good in this, but I lost it all after my son passed away*.' (S1)
Q12. could do the things they wanted to do?	"*She hasn't wanted to do much. Like I said, normally she would be really wanting to go to church, but she hasn't expressed that desire… She hasn't been saying she's missing out on anything or wanting to do anything differently, or wanting to do the things that she was doing before the fall (last month)*." (P10)
**Use of the recall period in retrieval**	
**Strategy 1: Extending the recall period to capture significant events**	
Q3. felt exhausted?	"*If I was answering only occasionally, that would be maybe once a week. Maybe once every 10 days*." (J5)

Q4. felt lonely?	"*It's hard to tell (if she felt lonely), but if it was the last 14 days I can tell you a very interesting occurrence. But I would say she feels lonely sometimes. Not often because I try to be here, but she's definitely lonely*." (P3)
Q5. had trouble concentrating or thinking clearly?	"*I have seen it increasing rapidly. There are some other card games we play, and even there his concentration has changed quite a lot in the recent period. It's difficult for me to say clearly whether seven days or two weeks, but I can see that it is declining… As far as bridge is concerned, over a couple of years. Other cards, maybe over the last month or so*." (S1)
Q6. felt anxious?	"*(He's had anxiety) every say once every three months… Generally, it's too because he is very concerned about something could be happening with a family member that he becomes quite concerned and worried*" (J17)
Q7. felt sad or depressed?	"*I can't distinguish if it's depression which is making her feel that way to begin with. Which is a big possibility because her life has changed so drastically from when six days she used to be with me, and the seventh day was Sunday which was a half day, because we used to be out to now being stuck in the house. So depression could be also a part of it, I'm not too sure."* (J7)
Q10. felt accepted by others? (e.g. felt like they were able to be themselves and that they belonged)	"*The last seven days we didn't have much of an opportunity to talk about (these feelings and thoughts) … I would still assume that he feels that way*." (S1)<br><br>"*I wish this was a little bit long, the last fourteen days. I'm going to use the event – I took Mum to her old scholar's function, and this one I decided to attend, because she asked me to… It went down like a house on fire. Mum socialised, and she went around, because I was there, and I got her up and made sure she talked to everyone, and she moved around the table, and it went down really well. So, I would say she felt accepted by others, felt like they were able to be themselves and that they belonged. I'd like to say often, but I'm going to say sometimes*." (P3)
Q11. felt good about themselves?	"*(I'll) probably take that maybe to the last nine or ten days. Mum often will say to me, 'What can I do to repay you? I appreciate everything you're doing, I'd like to be able to do something for you*.'" (P14)
**Strategy 2: Drawing on the past to contextualise recent behaviours**	
Q1 getting around inside and outside? (using, for example, a walking stick or wheelchair if they normally use them)	"*She's very slow, she has lost a lot of awareness of assessing her environment. That's why I would call it some difficulty and not slight difficulty. Different to what happened say six months ago. Six months ago, I would have said slight difficulties, now I would say some difficulties*." (P13)<br><br>"*I focused specifically on the last seven days… about three months ago we gave her a shot of cortisone in one of her hips, and it (still) really limit her ability to get around.*" (P3)

Q2. doing day-to-day activities? (e.g. working, shopping, housework)	"*I can compare it to before, when she was admitted to hospital. If I came over with my husband and did what we've been doing this past week – like cleaning the house, getting things in order, doing her laundry – she wouldn't have wanted us to do it really, she would have been up and round trying to stop us, but she hasn't at all this past week. That's been a marked difference.*" (P10)<br><br>"*Before all this happened, I used to take her out on Saturdays, get her to do her nails, go for a movie, walk around the mall and come back. The whole day we used to be out… now she can't do the whole day. Now it's like a two hour thing, max, she starts getting tired and we are back home*" (J7)
Q3. felt exhausted?	"*Ten years back, he was playing tennis regularly, he was playing badminton, and some 30 years back he was a good cricket player. So he was quite an active sportsperson. He enjoyed his sport. And even now, when he does bike, he is quite happy. Just that he forgets that he has done it and sometimes he does it more often, but he's quite happy when he is on his bike.*" (S1)
Q4. felt lonely?	"*He doesn't actually say he's lonely. He'll say it in a different way, that he's wanting to fix his flaws, and he's wanting to get back into shape.*" (J17)
Q5. had trouble concentrating or thinking clearly?	"*It's been fairly constant for quite a few months. The last seven days it has been worse, yeah. But prior to that, for the last 12 months it was very hard to watch a movie with him, because he'd forget who the people were because he couldn't concentrate on you know all the movements and things that happen in a movie.*" (P12)
Q6. felt anxious?	"*He just broke into song and started singing, and that's the kind of thing he does. When he's feeling anxious… he'd just break into song. That was his way of deflecting what was happening.*" (J18)<br><br>"*When he first moved in with us, he was anxious a lot because he kept saying he had to go back to his house but he couldn't remember where his house was. That went on for a few months and that was last year. But now he's just accepted that he's here with his son and [NAME] always reassures him that he's safe when he was going through all that anxiety at the beginning and that hasn't happened for over 12 months. That was the only thing that was causing him anxiety.*" (J15)
Q7. felt sad or depressed?	"*Most of the time. Before Dad was diagnosed with dementia, Dad had suffered with anxiety and depression for around 40-odd years. Maybe a bit more.*" (S11)<br><br>"*History-wise, I think it's pretty normal, being sad about my dad. That's a pretty normal thing. She doesn't really dwell on it. I think she's a lot more anxious than depressed.*" (P8)

Q8. felt they had no control over their day-to-day life? *(e.g. had no choice to do things or have things done for them as they liked and when they wanted)*	"*Well, I can compare it to before, when she was admitted to hospital. If I came over with my husband and did what we've been doing this past week, like cleaning the house, getting things in order, doing her laundry, she wouldn't have wanted us to do it really, she would have been up and round trying to stop us, but she hasn't at all this past week. That's been a marked difference. She's just let us go about our business. Whereas, before, she would have wanted to control that a bit more. The last seven weeks or since she's been discharged, she's shown very little interest in actually what we've been doing. She's just been sitting in front of the TV, quite happily, content.*" (P10)
Q9. how much physical pain you think the person had	"*If I look at the last four days, she has not mentioned any pain at all whereby before she used to just about every day say, 'Oh it's just sore there' and I'd go, 'Do you want some Panadol?'. 'Oh no, no, I don't want any tablets'. That's why I was thinking mild. When she had an attack of her gallbladder pain (years ago), that was very severe physical pain, and I had to take her to the hospital.*" (P13)
Q10. felt accepted by others? (e.g. felt like they were able to be themselves and that they belonged)	"*Let's say six months ago, he felt like he belonged because he had a few people that he would be able to watch a movie with and sit with and feel okay with, and now that's gone because of their declining. One's declining faster than Dad, but the other one is now frightened of Dad because of Dad's behaviour. Now, his feeling of belonging, there is none, unless the family are there.*" (S11)
Q11. felt good about themselves?	"*Most or all of the time. I don't think there's even an often. I think overall, that's just her personality.*" (P8)
Q12. could do the things they wanted to do?	"*Here's things like we've been sorting through a bunch of material at home and he's finding that even trying to read through or look through some of those things, it's very limited. Before, he would have just done it really quickly. Or there's problems that he's got with his computer and there's things he would like to do with that, that's just not a possibility anymore.*" (P19)
**Proxy perspectives**	
Challenging to answer items about constructs that were perceived as less observable or expressed	"*He's never alone. But in saying that, just through my job I know that just because someone's with you, doesn't mean you're not lonely. But I just think it's harder go gauge when someone – you equate being alone with being lonely. But you could still be with a lot of – I always say – we've got 15 people in a cottage at my work, and it's a busy little cottage, but you can still be very lonely. But I would say my dad, with what I observe, I'd say no.*" (J18)<br><br>"S*ometimes you can be sitting with someone and chatting away and you could still feel lonely. Obviously, if I ask him if he felt lonely, he would say 'no', whether it was true or not. He doesn't open up to me that way.*" (P2)

	"*I would say often he feels lonely, but it's not something that he expresses to me. As in, he doesn't just go, "I'm lonely." It's more of a he'll call me and he'll say, "What are you doing?" I'll say to him, "I'm at work," or wherever I am. He'll say, "I thought you just might pop over if you're going past. I feel like maybe we can do some painting or something." That tells me that he's looking for connection.*" (S11)<br><br>"*I'd like to say often (she feels accepted), but I'm going to say sometimes… I felt the ladies in the club, some of them might have treated Mum differently because she suffered this condition… they talk about Mum like she's no longer here."* (P3)<br><br>"*I think that would be the same whether you had dementia or not. Who sits around and says, "I feel accepted by others. I feel I belong here." It's such an internal gauge and a subjective feeling. It's so fluid as well. Not taking my mother, for example. Just taking the general population. So, let's use myself as an example. That might change from minute to minute, if I was in a group of people and I was doing something – one minute I might do this and I might say something and I might go – I don't know. This is such a subjective and esoteric question.*" (J5)<br><br>"*I think there are times when he feels I'm a little bit bossy. He hasn't got perhaps as much control as he would like. But then if he says to me, 'Look, I can do this myself,' and I say, 'Fine, go ahead,' then he has had some control over his life, hasn't he, because he explained to me that he wants to do it himself and does.*" (P9)
Supplementing response with proxy-patient perspective	"*She fought the disease for such a long time, and that's why she did bridge and online computer games, memory games. So, I don't think she's depressed, but I think she's sad that she lost the fight.*" (P3)<br><br>"*I'm answering these based on what [NAME] would say. I mean, at some places I definitely had to use my imagination, but most of the time I was stepping myself in shoes of [NAME], and thinking about what he would say if he was able to understand you and answer these questions… Questions like anxiety, I had to use more imagination. But I would say a large majority of questions – almost 80/85% of the questions – I think now that we share so many things with each other, I am able to read his mind. That's my guess, I could be wrong. But I'm able to read his mind.*" (S1)<br><br>"*I think it was a mixture of both (perspectives), depending on the question. Yeah. Because some of the things like – that's not tangible, like 'felt lonely', you can only go by what you're seeing. But that doesn't – so they might be presenting really well, but inside they're lonely. So to answer that – and if you asked him – if he was to do this, he would go, "no, I'm not*

	lonely." And I think no matter who does this (questionnaire), like everything I suppose, with people living with dementia, you have to know the person. To get to the bottom of what their needs are, you've got to know the person." (J18)
Concern that reduced insight could impact PLWD's responses	"*It would be hard for him to make sense of some of these questions and know how and what that means for him.*" (J17)<br><br>"*I don't know whether she feels that she has no control over her life. I don't think she does feel this because she still thinks she can do everything; she's told me 'I can go on a holiday' and I said, 'well who would look after you?' 'Oh, I can look after myself.'*" (P13)<br><br>"*He could be in more pain than what he's telling me, but he doesn't know how to express that or has the language for that.*" (S11)<br><br>"*I think (at the) core of her is she feels good about herself, but the anxiety comes in and is a part of that. Yeah, actually, that's a good question. I think maybe she probably questions herself as well. I don't know. Maybe. What about herself? I think maybe often then, because that anxiety is quite significant there. She questions herself and questions her judgement and questions. That's with the anxiety.*" (P8)
**Use of recall in answering strategies**	
Q1 getting around inside and outside? (using, for example, a walking stick or wheelchair if they normally use them)	"*In the last seven days, one night we needed to use the wheelchair because I couldn't get her to walk with the walker. That was one of those morphine nights which she was just totally off. But otherwise, I would think she's the same. I would say no difficulty because she is supported with the walker and she manages.*" (J7)
Q2. doing day-to-day activities? (e.g. working, shopping, housework)	"S*ome days it's okay, he might get three or four of the (tasks) down. Like the knives and forks and serviettes, but forget the salt and pepper. And, but you know, the last two days it's been almost every implement has to be instructed… I'd still say it's a lot of difficulty.*" (P12)<br><br>"*If I look at (question 2) from a non-physical side of things, I would say a lot of difficulty… Physically, it's still just slight… I'd probably say 'some difficulty,' so in between the two. If I'm putting it as an average, I would say he has some difficulty.*" (S11)<br><br>"*If you're looking at no difficulty to unable, you might have a scale of say one to five, and no difficulty is pretty self-explanatory and unable is pretty self-explanatory as well. And slight difficulty might be 25% of the time in the last seven*

	days there's been difficulty in doing something possibly, and then 50%, 75%, I don't know…. And then that would weight differently depending on what it was because some things have a higher impact on your ability to function and your quality of life than other things. So being able to actually cook your own meal as opposed to getting your mobile phone and your TV remote confused, they've got different implications for someone in their life." (P14)
Q3. felt exhausted?	"*Tired all of the time but exhausted for him would be if I average it out over the seven days, I would say he feels exhausted, yeah, quite often.*" (S11)
Q4. felt lonely?	"*I would say 'most, or all of the time', because during the day, again, days are great. I think she feels safe because there are people awake and in front of her, with the care workers, with us working. Even when we are working from home, we are literally it's like she's on the lounge and we are at the table there so she can constantly see us, and the TV is there.*" (J7)
Q5. had trouble concentrating or thinking clearly?	"*It ranges all the time that she is with us, but she only exhibits that problem after lunch for an hour or two. It is not representative of the whole week… However, because of the consistency of the Sunday, I would anticipate that that is a common occurrence. But I cannot say definitively that that is the case every single day.*" (P16)
Q6. felt anxious?	"*I would say occasionally. Because last week when he wanted to go to the party but couldn't, he was very anxious. (Occasionally is) when having to do something out of the ordinary. It's not a daily – again, that's another thing, it's not so real, tangible.*" (J18)
Q7. felt sad or depressed?	"*If I really call that depressed too is how long does this last too? If someone says it and is quiet for a little while and then eventually gets up and does things or starts talking or has some enjoyment again, I wouldn't call that really – you could be feeling sad. I think sometimes what I think I put in there too is how long something is before I call it depressed… I've never seen her depressed all day or long periods of time. It's really more that she says 'I'm feeling a little flat' and then I say 'okay, let's maybe' – I know what she loves doing and what gives her joy and I try that, we can do something, we go walk around the property or we play ball with the dogs or do something. She loves being outdoors and in the sunshine.*" (P13)<br><br>"*Most or all of the time... At least four days out of the seven would be a good week. But when he has a support worker come and they take him out, he still does do that.*" (J17)
Q9. how much physical pain you think the person had	"*My mum is very stoic, so the fact that she is saying something about the pain indicates that it's there. I think if it was severe we would be hearing about it every day, but we're not… I think it qualifies as moderate because it's interrupting her sleep.*" (P10)

	"*If I was answering this in a situation to a health professional or someone like that, what I might classify as mild physical pain might differ from – it's a little bit subjective unless there's actually using some kind of pain scale… It's defining in your questions, what you mean by mild physical pain. So, mild physical pain might be – I don't know, two or three on a pain scale. Moderate physical pain might be five. Severe might be eight or nine so that the response that you're trying to ascertain, so that the person answering the question can understand to give you the best accurate data.*" (J5)
Q11. felt good about themselves?	"*She's seemed content, but does she feel good about herself? I really don't know. I don't know that I could confidently answer this in any way. She doesn't seem unhappy, she doesn't seem troubled. If anything, I would lean towards the positive, that she has felt good about herself. I don't know what dementia does to someone's self-reflection… I'll go the middle, sometimes to be safe, but I haven't seen evidence either way, so I will stay neutral. I can only give you a neutral answer and say 'sometimes'.*" (P10)
Q12. could do the things they wanted to do?	"*That probably goes sometimes again because none of the time really wouldn't be true because there is lots of things that she – if she wants to do something and it's possible to do that, I encourage her to do this and do things with her. I always ask her what she wants to do, I try to get her involved. Not all of the time because sometimes the requests are stuff that I just know she cannot do 'cause she's a danger to herself if she did it.*" (P13)
